# The Achilles’ heel of the Trojan Horse? A systematic evaluation of cefiderocol susceptibility testing

**DOI:** 10.1093/jac/dkaf391

**Published:** 2025-10-21

**Authors:** Deny Tsakri, Stefanos Ferous, Ioannis Baltas, Louis Grandjean, Cleo Anastassopoulou, Athanasios Tsakris

**Affiliations:** Department of Microbiology, Medical School, National and Kapodistrian University of Athens, Athens, Greece; Department of Microbiology, Medical School, National and Kapodistrian University of Athens, Athens, Greece; Infection, Immunity & Inflammation Department, UCL Institute of Child Health, London, UK; Infection, Immunity & Inflammation Department, UCL Institute of Child Health, London, UK; Department of Microbiology, Medical School, National and Kapodistrian University of Athens, Athens, Greece; Department of Microbiology, Medical School, National and Kapodistrian University of Athens, Athens, Greece

## Abstract

Cefiderocol, an innovative siderophore cephalosporin, presents a novel therapeutic option against a spectrum of multidrug-resistant (MDR) Gram-negative pathogens. Nevertheless, resistance remains a formidable challenge, particularly among metallo-beta-lactamase (MBL)-producing organisms. Accurate antimicrobial susceptibility testing (AST) for cefiderocol is complex due to the labour-intensive broth microdilution (BMD) reference method requiring iron-depleted media, lacking reproducibility. In response, commercial AST methods, including BMD panels, disc diffusion (DD), and gradient diffusion test, have been developed. Commercial BMD panels, such as ComASP^®^ and UMIC^®^, demonstrate potential, with the latter reaching categorical agreement (CA) above 90%. Yet, essential agreement (EA) remains between 75% and 85%, below the 90% desired threshold, with very major errors (VMEs) occurring frequently (∼15%). Disc diffusion (DD) methods, while practical, often overcall resistance, leading to major errors (MEs) with a median across studies of 29%. Among disc manufacturers, MASTDISCS^®^ performed best, with a pooled CA of 93.2%, 5.4% ME and 6.3% VME. Overall, discs recorded a CA of 79.4%, MEs of 29.0% and VMEs of 13.9%. Gradient diffusion tests performed least favourably among all methods, exhibiting a notably high VME rate of 41.1%, and their use should be limited. Cefiderocol AST is further complicated by disparities between European Committee on Antimicrobial Susceptibility Testing (EUCAST) and Clinical and Laboratory Standards Institute (CLSI)/Food and Drug Administration (FDA) breakpoints, contributing to inconsistencies in susceptibility categorization across laboratories. Additional challenges, such as trailing endpoints and microcolonies within inhibition zones, further confound readings, especially in DD assays. Consequently, the standardization and rigorous validation of the best performing cefiderocol AST methodologies are imperative to ensure reliable susceptibility outcomes and optimized outcomes for patients with MDR infections.

## Introduction

The emergence of multi- and pan-drug-resistant Gram-negative bacteria worldwide represents a significant threat to public health.^1^ Among the most frequently used novel combinations in clinical practice are three beta-lactam/beta-lactamase inhibitor combinations: meropenem-vaborbactam, ceftazidime-avibactam and imipenem-cilastatin/relebactam.^1^ These combinations primarily target serine-β-lactamase-producing carbapenem-resistant Enterobacterales (CRE).^1^ However, therapeutic options for infections caused by metallo-beta-lactamase (MBL)-producing Enterobacterales, carbapenem-resistant *Acinetobacter baumannii* (CRAB) and difficult-to-treat *Pseudomonas aeruginosa* (DTR-*P. aeruginosa*) remain limited.^2^ Treatment often relies on combination therapies involving older, highly toxic antibiotics such as colistin, aminoglycosides, tigecycline and fosfomycin.^2^

Cefiderocol, a novel, siderophore fifth-generation cephalosporin, has recently been approved for the treatment of hard-to-treat Gram-negative infections, including those caused by MBL-producing Enterobacterales, CRAB and DTR-*P. aeruginosa.*^3,4^ Its use is becoming more widespread in clinical practice.^3,4^ However, due to its recent introduction, real-world data on cefiderocol’s efficacy are still limited, with most studies focusing primarily on CRAB infections. Cefiderocol has been evaluated in three multicentre randomized trial: one Phase 2 trial, APEKS-cUTI, and two Phase 3 trials, CREDIBLE-CR and APEKS-NP.^5^ Additionally, there have been discrepancies in the accuracy of current antimicrobial susceptibility testing (AST) methods for cefiderocol.^6^

The aim of this review is to provide an overview of the current use of cefiderocol, with a focus on real-world evidence regarding its efficacy and safety. It also intends to address various issues and challenges surrounding susceptibility determination.

## Methodology and strategy

To conduct this review, a comprehensive literature search was performed using Ovid MEDLINE, employing the search terms ‘Cefiderocol’, ‘Fetroja’ or ‘S-649266’ in abstract or title to identify relevant studies published up to 23 April 2025. Articles were eligible for inclusion if their abstracts or keywords contained the terms ‘disc’, ‘disk’, ‘broth microdilution’, ‘agar dilution’’, ‘gradient diffusion’, ‘etest’, ‘strip’, ‘testing method’, ‘test method’ or ‘susceptibility testing’. For each study evaluating the performance of commercially available tests according to European Committee on Antimicrobial Susceptibility Testing (EUCAST) guidelines, data were extracted and recorded in an Excel spreadsheet, capturing metrics such as categorical agreement (CA), essential agreement (EA), major error (ME), very major error (VME) and bias. Weighted averages were calculated for each performance metrics across all commercially available methods to ensure a comprehensive synthesis of the data.

## Cefiderocol: siderophores and the ‘Trojan Horse’ strategy

Siderophores are iron-chelating molecules that bacteria use to scavenge iron from environment.^7^ When antibacterial agents attach to siderophores, they form sideromycins, which hijack the bacterial cell’s normal iron-uptake mechanism.^8,9^ This allows the antimicrobial compound to be delivered into the bacterial periplasm.^9^ This mechanism of action is often referred to as the ‘Trojan Horse strategy’.^9^ An example of a naturally occurring sideromycins is Albomycins, which are isolated from *Actinomyces subtropicus*.^10^ Albomycins consists of two parts: a siderophore that aids in the molecule’s uptake and an antibacterial component that inhibits bacterial tRNA synthetase—similar to the action of the antibiotic mupirocin.^11^ Albomycins exhibit a broad antibacterial spectrum, particularly against Gram-positive bacteria such as methicillin-resistant *Staphylococcus aureus* (MRSA) and *Streptococcus pneumoniae.*^12,13^ They may also target some Gram-negative bacteria, although they are ineffective against *Proteus* spp. and *Morganella* spp.^12,13^ Data on the detailed Gram-negative spectrum are limited, and there is no available information on the efficacy of Albomycins against CRE.^12,13^

Cefiderocol’s structure is similar to that of Albomycins, containing a catechol side chain that functions as a siderophore, along with a cephalosporin moiety closely related to cefepime and ceftazidime.^14^ Cefiderocol enters bacterial cells via passive diffusion through porins, as well as through iron-uptake mechanisms, primarily TonB-dependent iron transporters, found in the outer membrane of Gram-negative bacteria.^15,16^ This novel cell entry mechanism enables cefiderocol to resist hydrolysis by most beta-lactamases, including MBLs and OXA-carbapenamases.^17^ Notably, cefiderocol is effective against a broad range of Gram-negative bacteria, including CRAB, CRE and DTR-*P. aeruginosa*.^18,19^

## Cefiderocol: mechanisms of resistance—the role of beta-lactamase inhibitors

The unique structure of cefiderocol, along with its ability to hijack bacterial iron-uptake systems, helps explain its broad effectiveness against carbapenem-resistant Gram-negative bacteria. These bacteria often possess a wide range of resistance mechanisms, including carbapenemases, mutations in porins and efflux pumps and alterations in penicillin-binding proteins (PBPs). However, similar resistance mechanisms have been evolved in these bacteria to combat cefiderocol. The resistance mechanisms appear to be complex and interrelated, primarily involving a reduction in cefiderocol uptake (due to mutations in iron-uptake systems) and the production of carbapenemases.

Carbapenemases are enzymes that hydrolyse carbapenems, rendering them ineffective. There are two main types of carbapenemases: serine carbapenemases, which contain a serine residue in their active site, and MBLs, which use a zinc atom in their active site to cleave the antibiotic. The most common serine carbapenemases are from the KPC and OXA families, while frequently encountered MBLs include NDM, VIM and IMP enzymes. KPC carbapenemases are commonly found in Enterobacterales, while MBLs are isolated from both Enterobacterales and DTR-*P. aeruginosa*. Different OXA enzymes are produced by various carbapenem-resistant Gram-negative bacteria, with OXA-48 often seen in Enterobacterales in certain regions, and OXA-23 and OXA-51 being most commonly identified in CRAB.

The structure of cefiderocol allows it to remain stable in the presence of many carbapenemases, as shown by *in vitro* studies across numerous carbapenemase-producing isolates.^20,21^ Research by Poirel *et al.* and Ito-Horiyama *et al.* demonstrated cefiderocol’s stability when exposed to pure carbapenemase solutions, which is largely attributed to its chemical structure, including side chains such as the pyrrolidinium ring attached to the catechol moiety.^22–25^ Additionally, cefiderocol’s dual mode of entry into the periplasmic space might also result in high intracellular concentrations that are sufficient to overcome carbapenemase hydrolytic activity.

Despite these advantages, carbapenemases can influence cefiderocol’s minimum inhibitory concentrations (MICs) and contribute to resistance. MBLs, in particular, have been linked to reduced cefiderocol susceptibility, especially when combined with extended-spectrum beta-lactamase (ESBL) production and downregulation of siderophore receptors.^26^ Moreover, the presence of NDM enzyme, which has a higher affinity for cefiderocol than other MBLs, may favour the emergence of cefiderocol-resistant strains with mutations in the cirA receptor.^27^

Current research suggests that carbapenemase production can increase cefiderocol MICs, but it is rarely the sole factor responsible for resistance.^28^ In a study by Yang *et al.*,^28^ carbapenemase production (NDM and KPC) combined with mutations in iron-uptake systems (*cirA, envZ*) resulted in significant cefiderocol resistance. However, carbapenemase production alone is not associated with cefiderocol MIC above the clinical breakpoint. OXA-48, for example, caused only a small increase in cefiderocol MICs, even in the presence of iron-uptake mutations.^28^ In a separate study by Kocer *et al.*,^29^ the majority of cefiderocol-resistant *P. aeruginosa* isolates showed resistance due to mutations in iron-uptake systems, rather than carbapenemase production.

Interestingly, a study by Yamano *et al.*^30^ found that adding avibactam, a beta-lactamase inhibitor, reduced the MICs of cefiderocol-resistant CRAB isolates. This suggests that serine beta-lactamases, especially when combined with other resistance mechanisms, play a significant role in cefiderocol resistance.^30^ One specific isolate with an MIC of >32 μg/mL, did not harbour any *piuA* mutations but contained numerous beta-lactamase genes.^30^ However, the addition of avibactam restored cefiderocol susceptibility.^30^ Similarly, in the study by Kocer *et al.*,^29^ one isolate was resistant to cefiderocol solely due to overproduction of NDM. Inhibiting this enzyme restored cefiderocol activity.^29^ Furthermore, Asrat *et al.*^31^ supported these findings in their study of cefiderocol-resistant *A. baumannii.* These isolates were characterized by overexpression of the AmpC beta-lactamases (primarily ADC-25 and ADC-23) in combination with mutations in iron-uptake systems (primarily pirA and piuA). The addition of avibactam restored susceptibility, even when iron-uptake mutations were present.^31^

Although cefiderocol resistance is rare, understanding the interplay of beta-lactamases and iron-uptake systems is crucial. This knowledge may influence how cefiderocol is used, potentially promoting its use in combination therapies with beta-lactamase inhibitors. Gill *et al.*^32^ explored the efficacy of cefiderocol combined with ampicillin-sulbactam and ceftazidime-avibactam in a murine model of *A. baumannii* infections. Both cefiderocol-resistant and cefiderocol-susceptible isolates were tested.^32^ The combination of cefiderocol with beta-lactam/beta-lactamase inhibitors showed a synergistic effect, improving activity against cefiderocol-resistant isolates and reducing the emergence of resistance in highly susceptible isolates.^32^ However, no such effect was observed when cefiderocol was paired with meropenem, which lacks a beta-lactamase inhibitor.^32^

Other studies have also supported the use of cefiderocol with beta-lactamase inhibitors.^33^ For this reason, cefiderocol in combination with xeruborbactam, a novel inhibitor targeting both MBLs and serine carbapenemases, is currently under investigation in early clinical studies.

## Disparities in cefiderocol breakpoint criteria: EUCAST, CLSI and FDA perspectives

Significant challenges in the interpretation of AST results for cefiderocol arise from discrepancies in the interpretive standards set by the EUCAST, the Clinical and Laboratory Standards Institute (CLSI) and the US Food and Drug Administration (FDA).^34^ In April 2020, EUCAST introduced cefiderocol MIC and zone diameter (ZD) breakpoints for Enterobacterales and *P. aeruginosa*.^35^ However, breakpoints for *Acinetobacter* spp. and *Stenotrophomonas maltophilia* are still pending, and EUCAST currently recommends using general, non-species-specific pharmacokinetic/pharmacodynamic (PK/PD) breakpoints for these organisms.^35^ Matuschek *et al.*^36^ validated the susceptible ZD for PK/PD breakpoints at ≥17 mm for *A. baumannii* and ≥20 mm for *S. maltophilia*. Meanwhile, CLSI has set breakpoints for all the aforementioned microorganisms, but the FDA has not yet established breakpoints for *S. maltophilia.*^37,38^

Additionally, EUCAST has designated an area of technical uncertainty (ATU) for disc diffusion (DD) testing.^35^ This is defined as a ZD of 21–23 mm for Enterobacterales and 20–21 mm for *P. aeruginosa.*^35^ Within the ATU zone, if no alternative confirmatory method such as the laboratory broth microdilution (BDM) method is available, EUCAST recommends disregarding the ATU zone and using the established breakpoints for interpretation.^35^ While CLSI and the FDA recognize an intermediate susceptibility category, EUCAST deems these values as resistant for Enterobacterales.^35,37,38^ For *P. aeruginosa*, however, the FDA’s intermediate MIC value aligns with EUCAST breakpoint, whereas the FDA’s DD intermediate values (13–21 mm) partially overlap with the EUCAST ATU (20–21 mm), but fall below the resistance threshold (<22 mm).^35,37,38^

Breakpoints established by the three entities are presented in Table 1. The distinctions between EUCAST and CLSI/FDA are clearly delineated. A recent study by Baltas *et al.*,^39^ which evaluated cefiderocol susceptibility in 60 NDM-producing Enterobacterales, reported a resistance rate of 69.7% when results were interpreted using EUCAST breakpoints. In contrast, when CLSI/FDA breakpoints were applied, the resistance rate dropped to 4.5%.^39^ This underscores the discrepancy between European (EUCAST) and American (CLSI/FDA) susceptibility testing standards (Table 1).

**Table 1.** Breakpoints for cefiderocol MIC and ZD established by EUCAST, CLSI and FDA

Bacteria	EUCAST						CLSI						FDA					
	MIC (μg/mL)			ZD (mm)			MIC (μg/mL)			ZD (mm)			MIC (μg/mL)			ZD (mm)		
	S	ATU	R	S	ATU	R	S	I	R	S	I	R	S	I	R	S	I	R
Enterobacterales	≤2	—	>2	≥23	21–23	<23	≤4	8	≥16	≥16	9–15	≤8	≤4	8	≥16	≥16	9–15	≤8
*P. aeruginosa*	≤2	—	>2	≥22	20–21	<22	≤4	8	≥16	≥18	13–17	≤12	≤1	2	≥4	≥22	13–21	≤12
*Acinetobacter* spp.	—	—	—	—	—	—	≤4	8	≥16	≥15	—	—	≤1	2	≥4	≥19	12–18	≤11
*S. maltophilia*	—	—	—	—	—	—	≤1	—	—	≥15	—	—	—	—	—	—	—	—

## Evaluating diagnostic platforms for a novel siderophore cephalosporin

The unique mechanism of action of cefiderocol, which necessitates an iron-depleted environment, creates challenges in cefiderocol AST.1 The reference method for cefiderocol AST is BMD using iron-depleted-cation-adjusted Mueller–Hinton broth (ID-CAMHB), which ensures that MIC values accurately reflect *in vivo* activity for clinical breakpoints and PK/PD evaluation.^36^ However, preparing ID-CAMHB and conducting BMD are complex and burdensome tasks for clinical laboratories.^34^ To address these challenges, alternative commercially available AST methods, such as BMD panels, DD and gradient diffusion tests, have been developed to simplify cefiderocol testing.^34^ Several studies have assessed the performance of these methods across various bacterial species, using ID-CAMHB BMD as the reference method and applying EUCAST clinical and PK/PD breakpoints to evaluate MIC results.

## Commercial BMD panels for cefiderocol susceptibility testing

### Evaluation of the Sensititre EUMDROXF^®^ panel

The first BDM panel developed for cefiderocol testing was the Sensititre EUMDROXF^®^ panel (Thermo Fisher Scientific, Waltham, MA, USA). This panel incorporates cefiderocol and an iron chelator within its wells, allowing for the rehydration of all wells, including those containing cefiderocol, using conventional CAMHB.^34^ Cefiderocol concentrations in the EUMDROXF^®^ panel range from 0.03 to 8 mg/L, and the panel can be stored at temperatures between 15°C and 25°C.^40^

Devoos *et al.*^41^ evaluated the performance of the Sensititre EUMDROXF^®^ panel by testing 150 clinical isolates of *P. aeruginosa*. When compared with the reference BMD method using ID-CAMHB, the Sensititre microplates tended to overestimate MICs.^41^ It also showed a CA rate below the 90% threshold and produced several VMEs.^41^ Another study, utilizing pre-prepared frozen (−80°C) Sensititre CML1FEUD plates (Thermo Fisher Scientific, Waltham, MA, USA) containing cefiderocol in ID-CAMHB as the reference method, assessed cefiderocol susceptibility in 100 CRE isolates.^42^ This study reported a higher CA, notable EA, and minimal ME and VME rates.^42^

Castillo-Polo *et al.*^40^ conducted cefiderocol AST on 104 Enterobacterales isolates, achieving a very high CA (99%) with only a few VMEs. Despite these improvements in Enterobacterales testing, the Sensititre EUMDROXF^®^ panel has shown inconsistent performance across studies, with a tendency to overestimate MICs and produce VMEs in some cases. Due to issues with the MHB, the Sensititre EUMDROXF^®^ microplates have been deemed unsuitable for determining cefiderocol susceptibility as of February 2022.^43^

### Performance analysis of the ComASP^®^ BMD panel

Another commercially available BMD panel is the ComASP^®^ microplate (Liofilchem, Roseto degli Abruzzi, Italy).^44^ This assay features a dual-panel format antibiotic concentrations spanning from ≤0.008 to 128 mg/L in 15 doubling dilutions.^40,43^ It requires storage at temperatures between 2°C and 8°C.^40^ The ComASP^®^ panel can be integrated into standard laboratory procedures by utilizing the same 0.5 McFarland bacterial inoculum as in conventional susceptibility testing, followed by a 1:20 dilution in saline.^43,45^

A 2023 study evaluating cefiderocol susceptibility in 97 *Acinetobacter* spp. isolates found that, when compared with the reference BMD method, the ComASP^®^ test met the 90% CA threshold for reliability.^44^ However, it demonstrated suboptimal EA rate and significant bias and resulted in four VMEs.^44^ In a related study, the CA was lower, accompanied by an elevated VME rate.^46^

Similarly, Kolesnik-Goldmann *et al.*^47^ identified notable VMEs when testing 100 *A. baumannii* isolates. Castillo-Polo *et al.*^40^ assessed the ComASP^®^ panel against Enterobacterales, noticing a low ME rate but a considerable upward bias. Stracquadanio *et al.*^6^ found high CA for Enterobacterales and a perfect CA for *P. aeruginosa*, though the performance for *A. baumannii* was variable, with the panel tending to underreport the elevated resistance.^6^ The EA ranged from 52% to 60%, depending on whether the trailing effect was disregarded or considered, respectively.

A study of 286 multidrug-resistant (MDR) Gram-negative isolates, including Enterobacterales, *P. aeruginosa* and *A. baumannii* confirmed consistent performance.^48^ The ComASP^®^ panel showed acceptable CA but fell short in EA and had a concerning VME rate. Its performance was highly variable across bacterial species, delivering reliable results for *P. aeruginosa* but exhibiting significant limitations for *A. baumannii* and Enterobacterales. Given the risk of missing resistant isolates, clinical reliability is compromised, necessitating cautious use and further validation.

### Performance metrics of UMIC^®^ panel

The latest BMD panel developed is the UMIC^®^ BMD strip (Bruker Daltonics GmbH & Co. KG, Bremen, Germany).^44^ They are individual BMD MIC strips containing pre-dried antibiotic, ranging from ≤0.03 to >32 mg/L, which have been proven effective for key therapeutic antibiotics, including colistin, piperacillin/tazobactam, daptomycin and others.^49^ The strips have a long lifespan, can be stored at ambient temperature and allow for testing of one strain at a time. They come with pre-prepared ID-CAMHB vials, and the standard 0.5 McFarland bacterial inoculum is used.^44,49^

Dortet *et al.*^49^ evaluated the UMIC^®^ strips across 283 Gram-negative bacteria, primarily carbapenemase producers. They found robust CA and EA, but with notable VMEs and a tendency to underestimate MICs.^49^ For 180 Enterobacterales, CA and EA were slightly lower, with elevated VMEs and a significant negative bias.^49^ Among 103 non-fermenters, including *P. aeruginosa*, *A. baumannii* and *S. maltophilia*, the UMIC^®^ strips demonstrated improved CA, particularly for *P. aeruginosa*, with acceptable EA, fewer MEs and VMEs and a minimal overall bias.^49^ However, one *S. maltophilia* isolate, which was susceptible by the reference method, was misclassified as resistant, constituting an ME.^49^

Jeannot *et al.*^44^ compared the UMIC^®^ strips to the reference BMD method against *Acinetobacter* spp. and found a CA rate of 93.8%, exceeding the 90% acceptability threshold, but the EA was only 78.4%, with four VMEs and a 42.3% tendency to underestimate MICs.

Further evaluation of the UMIC^®^ strips in *A. baumannii* showed a CA of 89% and an EA of 76%, both falling below the 90% threshold, with an inflated VME rate (37.5%).^47^ Bianco *et al.*^50^ demonstrated that the UMIC^®^ BMD strip performed reliably across 256 Gram-negative bacterial isolates, achieving high CA (>90%) for Enterobacterales, *P. aeruginosa*, *A. baumannii* and *S. maltophilia*.^50^ However, EA was lower, particularly for *S. maltophilia*, which also exhibited a higher VME rate.^50^ The strip showed low VME rates for the other species, but a notable ME rate for Enterobacterales.

Overall, the UMIC^®^ cefiderocol BMD strip performs well across Gram-negative isolates, with excellent CA for non-fermenters, alongside strong EA and absent VMEs for *P. aeruginosa.* It maintains comparable accuracy across Enterobacterales and non-fermenters, although some species occasionally show underestimated MIC values. Detailed performance metrics for each study are provided in Table 2.

**Table 2.** Comparative performance of commercially available BMD panels for cefiderocol MIC testing across Gram-negative isolates according to EUCAST breakpoints

Commercial BMD panels	Microorganism	No. of isolates	Study	CA (%)	EA (%)	ME (%)	VME (%)	Bias (%)
EUMDROXF^®^ (Thermo Fisher)	Enterobacterales	100	Bonnin *et al.*^42^	95.0	87.0	1.6	2.8	N/A
		104	Castillo-Polo *et al.*^40^	99.0	81.7	0	7.7	+2.6
		Weighted average		97.0	84.3	0.78	5.30	+2.6
	*P. aeruginosa*	150	Devoos *et al.*^41^	86.7	69.3	10.2	12.5	+68.2
	**Weighted average for all species**			**92.4**	**77.9**	**4**.**8**	**8**.**3**	**+41**.**3**
ComASP^®^ (Liofilchem)	Enterobacterales	47	Stracquadanio *et al.*^6^	97.0	66.0	0	2.0	+29.8
		104	Castillo-Polo *et al.*^40^	98.1	88.5	1.1	7.7	+27.9
		38	Bianco *et al.*^48^	92.1	84.2	6.9	11.1	+0.05
		Weighted average		96.6	82.0	2.0	7.0	+22.8
	*P. aeruginosa*	50	Stracquadanio *et al.*^6^	100	88.0	0	0	+24.0
		5	Bianco *et al.*^48^	100	80.0	0	0	+0.09
		Weighted average		100	87.3	0	0	+21.8
	*Acinetobacter* spp.	97	Jeannot *et al.*^44^	95.9	81.4	0	9.5	−7.2
		25	Stracquadanio *et al.*^6^	92.0	60.0	11.1	6.25	−47.8
		100	Kolesnik-Goldmann *et al.*^47^	88.0	76.0	6.6	29.2	N/A
		27	Pasteran *et al.*^46^	74.1	29.6	7.4	22.2	+47.4
		7	Bianco *et al.*^48^	100	85.7	0	0	+5.6
		Weighted average		90.2	71.9	4.4	18.0	−3.7
	**Weighted average for all species**			**85.9**	**78.4**	**3**.**03**	**11**.**8**	**+14**.**8**
UMIC^®^ (Bruker)	Enterobacterales	90	Bianco *et al.*^50^	91.1	81.1	14.0	2.5	+26.7
		180	Dortet *et al.*^49^	87.8	91.7	5.4	23.5	−25.0
		Weighted average		88.9	88.2	8.3	16.5	−7.8
	*P. aeruginosa*	21	Bianco *et al.*^50^	90.0	92.5	19.1	0	N/A
		49	Dortet *et al.*^49^	98.0	93.9	2.3	0	+12.2
		Weighted average		95.6	93.3	7.3	0	+12.2
	*Acinetobacter* spp.	39	Bianco *et al.*^50^	97.4	89.7	0	5.6	N/A
		44	Dortet *et al.*^49^	90.9	84.1	3.2	23.1	−11.4
		97	Jeannot *et al.*^44^	93.8	78.4	3.6	9.5	−25.2
		100	Kolesnik-Goldmann *et al.*^47^	89.0	76	2.6	37.5	N/A
		Weighted average		92.1	80.0	2.7	21.1	−20.9
	*S. maltophilia*	22	Bianco *et al.*^50^	96.7	73.3	0	16.7	N/A
		10	Dortet *et al.*^49^	90.0	89.3	10	0	N/A
		Weighted average		94.6	78.3	3.1	11.5	N/A
	**Weighted average for all species**			**91.3**	**84.7**	**5**.**5**	**16**.**5**	−**9**.**7**

CA, categorical agreement; EA, essential agreement; ME, major error; VME, very major error.

Overall values are presented in bold.

## Performance analysis of commercially available cefiderocol discs

Matuschek *et al.*^36^ determined that DD represents a reliable and straightforward technique for assessing cefiderocol susceptibility, although results within the ATU necessitate confirmation through BMD. For all studies, ID-CAMHB BMD served as the reference method, and EUCAST clinical and PK/PD breakpoints were applied to evaluate the ZD results.

### Evaluation of MASTDISCS^®^ performance in cefiderocol susceptibility testing

Morris *et al.*^34^ evaluated the performance of 30 μg cefiderocol MASTDISCS^®^ (Mast Group Ltd., Bootle, UK) and reported a high CA for Enterobacterales, with no VMEs. These discs also achieved perfect CA for *S. maltophilia*, while for *P. aeruginosa* and *A. baumannii* exhibited MEs (10% and 20%, respectively) and VMEs (25% and 11%, respectively).^34^ This suggests that the performance of MASTDISCS^®^ may vary among non–glucose-fermenting organisms.^34^

In an interesting study by Devoos *et al.*,^41^ the performance of MASTDISCS^®^ was evaluated on six different Mueller–Hinton agar (MHA) plates (Oxoid, Thermo Fisher Scientific; Mast Diagnostic; Becton Dickinson; I2a diagnostics; Bio-Rad; and bioMérieux). The best results were achieved when using Becton Dickinson MHA, where the VME rate was the lowest, and the CA reached 94.1% when strains within the ATU zone were excluded.^41^ However, across all MHA plates, the overall CA was 82.9%, with frequent microcolony observations.^41^ In a separate study using Becton Dickinson MHA, a similar CA was reported for *Acinetobacter* spp., accompanied by an elevated VME rate (42.9%).^44^

Bianco *et al.*^50^ found consistently high CA across all species tested, with only a single ME observed in Enterobacterales. A significant proportion of isolates fell within the ATU, particularly over a third of the strains, and a small fraction of these correlated with resistant MICs.^50^ A prior study by the same group highlighted that ATU results were prevalent among ceftazidime/avibactam-resistant KPC- and NDM-producing Enterobacterales.^48^ Liu *et al.*^51^ evaluated MASTDISCS^®^ on nearly 500 *A. baumannii* isolates using Oxoid MHA and achieved high CA with minimal VMEs. In a related study, high CA was observed with a small subset of susceptible isolates misclassified as resistant, but no VMEs were reported.^52^

Overall, MASTDISCS^®^ demonstrated robust performance across multiple studies, providing acceptable CA results. However, some variability was evident, especially with MEs and VMEs, indicating occasional discrepancies in susceptibility classification compared with the reference method. While the discs generally offer reliable results, their performance can be inconsistent across different species and testing conditions, particularly for non–glucose-fermenting organisms.

### Assessment of Liofilchem^®^ discs in cefiderocol susceptibility testing

The Liofilchem^®^ cefiderocol 30 μg disc (Liofilchem, Roseto degli Abruzzi, Italy) has been evaluated in several studies, with mixed results. In one study, performed on Becton Dickinson MHA, the disc showed optimal performance, obtaining a CA rate of nearly 90%.^41^ However, another study, employing the same MHA, reported a lower CA, with half of the resistant isolates classified as VMEs.^44^ Castillo-Polo *et al.*^40^ noted that when using Liofilchem^®^ disc on Oxoid MHA, the disc tended to overestimate the ZDs, leading to incorrect classification of almost 50% of the susceptible strains as resistant. A subsequent investigation revealed a considerable proportion of isolates falling within the ATU, which led to a substantial rate of MEs.^6^

Bianco *et al.*^50^ demonstrated a high CA for Liofilchem^®^ discs, with only a single ME and VME recorded for *A. baumannii*. However, a notable proportion of isolated fell within the ATU.^50^ A comprehensive study comparing DD on bioMérieux, Liofilchem and in-house-produced ID-CAMH agar plates yielded a CA slightly below the 90% threshold for *A. baumannii* compared with the reference BMD method.^47^ The ID-CAMH plates also exhibited a significantly reduced VME rate compared with the manufactured CAMH agar plates.^47^

Bonnin *et al.*^42^ evaluated 100 Enterobacterales isolates using Bio-Rad MH agar and reported a very high VME rate, though excluding isolates within the ATU reduced this rate considerably. Further research on *Acinetobacter* spp. indicated an unacceptable CA rate with an ME rate of approximately one in five tests but no VMEs, suggesting that while critical discrepancies were reliably detected, overall concordance was suboptimal.^46^ Other research highlighted a very low CA rate for Enterobacterales, with an extremely high incidence of MEs, alongside frequent inconsistencies in the ATU.^53^ For *P. aeruginosa*, the disc showed improved CA with fewer MEs, though VMEs were notably present.^53^ Another study displayed varied performance across tested species, with very similar performance for Enterobacterales but significantly elevated VMEs for *A. baumannii*.^54^ Finally, Bovo *et al.*^55^ reported a high CA with few MEs and no VMEs, achieving near-complete concordance when excluding strains within the ATU.

The Liofilchem^®^ disc exhibited moderate overall performance. While it achieved reasonable concordance in some studies, it showed varying results across different species. Its reliability was undermined by a high ME rate (40.9%), particularly for Enterobacterales, and a VME rate of 18.8%, with notably elevated VMEs for *Acinetobacter* spp. These findings highlighted that while the discs can be useful, their performance is inconsistent, especially for certain bacterial species, and is further complicated by frequent ATU-related discrepancies.

### Performance analysis of Oxoid^®^ discs for cefiderocol susceptibility

An alternative for DD susceptibility testing is the Oxoid^®^ 30 μg cefiderocol disc (Thermo Fisher Scientific, Waltham, MA, USA). A study revealed its superior performance on Becton Dickinson, bioMérieux and Oxoid MHA plates, attaining a mean CA rate of 84.7%.^41^ In contrast, another study demonstrated a CA of only 72.2%, with over half of the resistant isolates (27/42) erroneously classified as susceptible.^44^

In a separate study, numerous isolates were misclassified as resistant, resulting in an ME rate of 30.8%.^40^ Findings from Bianco *et al*.^50^ indicated that Oxoid^®^ disc achieved a high CA across the evaluated species, with only a single VME observed in *P. aeruginosa*.^50^ However, they also noted a significant ATU rate (40%) in this bacterium, with 68.7% of these isolates exhibiting resistance.

Overall, the Oxoid^®^ disc demonstrated generally reliable performance with a mean CA slightly below the acceptable threshold, accompanied by a notably high VME rate, particularly for *Acinetobacter* spp.

### Evaluation of HardyDisk™ accuracy in determining cefiderocol susceptibility

A study evaluating the 30 μg cefiderocol HardyDisk™ (Hardy Diagnostics, Santa Maria, CA) reported a moderate CA for Enterobacterales, with some MEs observed.^34^ On the other hand, HardyDisk™ achieved a perfect CA for *S. maltophilia,* with no MEs or VMES.^34^ However, performance was less consistent for *P. aeruginosa* and *A. baumannii*, both of which showed low CA, alongside elevated ME and VME rates.^34^

In another study, Potter *et al.*^56^ assessed the performance of HardyDisk™ on 97 Gram-negative bacteria, though only 37 isolates were compared with the reference BMD method. The results revealed high ME rates for both Enterobacterales and *A. baumannii*.^56^ The HardyDisk™ exhibited a moderate mean CA, but also notable ME and VME rates. However, given that only two studies were conducted with a limited number of isolates tested, the reliability of these findings may be lower compared with other discs that have been evaluated in a broader range of studies. This limited dataset warrants cautious interpretation of its performance, especially considering the variability observed across different species.

### BD BBL™ Sensi-Disc™ for cefiderocol susceptibility

There exists another cefiderocol disc, specifically the BD BBL™ Sensi-Disc™ cefiderocol 30 μg (Becton Dickinson). However, no studies have been documented evaluating its performance.^57,58^ The precise performance of each study for every disc and species is comprehensively presented in Table 3.

**Table 3.** Performance comparison of cefiderocol antimicrobial susceptibility discs by manufacturer according to EUCAST clinical and PK/PD breakpoints

Commercial discs	Microorganism	No. of isolates	Study	CA (%)	ME (%)	VME (%)
MASTDISCS^®^ (Mast Diagnostics)	Enterobacterales	90	Bianco *et al.*^50^	98.2	2.0	0
		58	Morris *et al.*^34^	90.0	14.0	0
		Weighted average		95.0	6.7	0
	*P. aeruginosa*	21	Bianco *et al.*^50^	100	0	0
		150	Devoos *et al.*^41^	82.9	N/A	N/A
		14	Morris *et al.*^34^	86.0	10.0	25.0
		Weighted average		85.1	4.0	10.0
	*Acinetobacter* spp.	39	Bianco *et al.*^50^	94.9	4.8	5.6
		14	Morris *et al.*^34^	86.0	20.0	11.0
		97	Jeannot *et al.*^44^	81.4	N/A	42.9
		468	Liu *et al.*^51^	97.0	N/A	1.9
		89	Uskudar-Guclu *et al.*^52^	97.8	3.7	0
		Weighted average		94.6	5.6	7.7
	*S. maltophilia*	22	Bianco *et al.*^50^	100	0	0
		11	Morris *et al.*^34^	100	0	0
		Weighted average		100	0	0
	**Weighted average for all species**			**93.2**	**5.4**	**6**.**3**
Liofilchem^®^ cartridge discs (Liofilchem)	Enterobacterales	100	Bonnin *et al.*^42^	77.0	1.6	56.4
		75	Bovo *et al.*^55^	92.0	16.7	0
		104	Castillo-Polo *et al.*^40^	57.7	48.4	0
		47	Stracquadanio *et al.*^6^	72.0	21.0	0
		90	Bianco *et al.*^50^	100	0	0
		84	Barth *et al.*^53^	32.1	90.3	4.5
		342	Nayak *et al.*^54^	38.6	89.7	0
		Weighted average		58.2	54.6	7.2
	*P. aeruginosa*	150	Devoos *et al.*^41^	84.5	N/A	N/A
		21	Bianco *et al.*^50^	100	0	0
		19	Barth *et al.*^53^	79.0	14.3	25.0
		24	Nayak *et al.*^54^	62.5	47.4	0
		Weighted average		83.1	22.0	7.4
	*Acinetobacter* spp.	39	Bianco *et al.*^50^	94.9	4.8	5.6
		20	Barth *et al.*^53^	85.0	21.4	0
		137	Nayak *et al.*^54^	58.4	17.4	66.2
		97	Jeannot *et al.*^44^	78.4	N/A	50.0
		100	Kolesnik-Goldmann *et al.*^47^	85.7	4.0	47.2
		27	Pasteran *et al.*^46^	81.5	22.2	0
		Weighted average		75.7	12.4	44.9
	*S. maltophilia*	22	Bianco *et al.*^50^	100	0	0
	**Weighted average for all species**			**67.2**	**40.9**	**18**.**8**
Oxoid^®^ (Thermo Fisher)	Enterobacterales	104	Castillo-Polo *et al.*^40^	73.1	30.8	0
		90	Bianco *et al.*^50^	100	0	0
		Weighted average		85.6	16.5	0
	*P. aeruginosa*	150	Devoos *et al.*^41^	84.7	N/A	N/A
		21	Bianco *et al.*^50^	95.8	0	6.3
		Weighted average		86.1	0	6.3
	*Acinetobacter* spp.	39	Bianco *et al.*^50^	94.9	4.8	5.6
		97	Jeannot *et al.*^44^	72.2	N/A	64.3
		Weighted average		78.6	4.8	47.5
	*S. maltophilia*	22	Bianco *et al.*^50^	100	0	0
	**Weighted average for all species**			**84.6**	**12.3**	**17**.**7**
HardyDisk™ (Hardy Diagnostics)	Enterobacterales	58	Morris *et al.*^34^	88.0	17.0	0
		8	Potter *et al.*^56^	75.0	40.0	0
		Weighted average		86.4	19.8	0
	*P. aeruginosa*	14	Morris *et al.*^34^	79.0	14.0	25.0
		10	Potter *et al.*^56^	100	0	0
		Weighted average		87.8	8.2	14.6
	*Acinetobacter* spp.	14	Morris *et al.*^34^	64.0	20.0	33.0
		13	Potter *et al.*^56^	76.9	23.1	0
		Weighted average		70.2	21.5	17.1
	*S. maltophilia*	11	Morris *et al.*^34^	100	0	0
		6	Potter *et al.*^56^	83.3	16.7	0
		Weighted average		94.1	5.9	0
	**Weighted average for all species**			**84.4**	**16.3**	**6**.**1**
BD BBL™ Sensi-Disc™ (Becton Dickinson)	N/A		N/A	N/A	N/A	N/A

CA, categorical agreement; ME, major error; VME, very major error.

Overall values are presented in bold.

## Evaluation of gradient diffusion testing for cefiderocol

The Liofilchem^®^ MIC test strip ((Liofilchem, Roseto degli Abruzzi, Italy) is currently the only commercially available gradient diffusion susceptibility testing method, with concentrations ranging from 0.016 to 256 mg/L.^6^ The supplier recommends utilizing these MIC test strips for *P. aeruginosa*, and they should be conducted on Becton Dickinson MHA.^42,44^

Stracquadanio *et al.*^6^ evaluated the gradient diffusion test on 50 *P. aeruginosa* isolates and reported a low EA despite achieving a perfect CA. This was due to the lower MIC values obtained compared with the reference BMD method.^6^ In a comparable study, the negative bias is once again emphasized, contributing to a VME rate of 50%.^41^ Another study conducted on Enterobacterales revealed poor performance, characterized by a VME rate exceeding 90%.^42^ Further research on KPC-producing Enterobacterales, despite achieving a CA at the threshold, confirmed the undervaluation of MICs, consequently leading to an increase in VMEs.^55^ Conversely, a different study reported a slight positive bias, with no VMEs, and only a few MEs recorded.^40^ For *Acinetobacter* spp., underestimation of the MIC was observed, accompanied by an elevated VME rate.^44^ Kolesnik-Goldmann *et al.*^47^ assessed the gradient diffusion tests across three different MHA plates, noting that in-house ID-CAMH plates had a VME rate that was reduced by more than half compared with the manufactured CAMH agar plates. In contrast, a separate evaluation on *Acinetobacter* spp. found lower CA with both ME and VME rates elevated.^46^ The performance of Liofilchem^®^ gradient diffusion test from all studies is presented in Table 4.

**Table 4.** Performance of the cefiderocol gradient diffusion testing Based on EUCAST breakpoints

Commercial MIC strips	Microorganism	No of isolates	Study	CA (%)	EA (%)	ME (%)	VME (%)	Bias (%)
Liofilchem^®^ MIC test strips (Liofilchem)	Enterobacterales	100	Bonnin *et al.*^42^	63.0	6.0	0	94.9	N/A
		75	Bovo *et al.*^55^	90.7	N/A	0	17.9	N/A
		104	Castillo-Polo *et al.*^40^	93.3	47.1	7.7	0	+2.9
		Weighted average		81.7	27.0	2.9	38.8	+2.9
	*P. aeruginosa*	150	Devoos *et al.*^41^	86.7	69.3	3.4	50.0	−30.4
		50	Stracquadanio *et al.*^6^	100	44.0	0	0	−52.0
		Weighted average		90.0	63.0	2.6	37.5	−35.8
	*Acinetobacter* spp.	97	Jeannot *et al.*^44^	76.3	59.8	0	54.8	−48.4
		100	Kolesnik-Goldmann *et al.*^47^	86.0	58.7	5.3	41.7	N/A
		27	Pasteran *et al.*^46^	66.7	18.5	22.2	38.9	+40.7
		Weighted average		79.4	54.3	5.0	47.0	−29.0
	**Weighted average for all species**			**83.4**	**48.2**	**3**.**5**	**41**.**1**	−**24**.**4**

CA, categorical agreement; EA, essential agreement; ME, major error; VME, very major error.

Overall values are presented in bold.

Based on the collective research conducted, it is evident that the Liofilchem^®^ MIC test strip should not be used, as it consistently yields lower MICs compared with the reference method and fails to identify resistant isolates. This could potentially lead to serious consequences in critically ill patients.

## Comprehensive assessment of the performance of commercially available cefiderocol susceptibility testing methods

**Figure 1.**
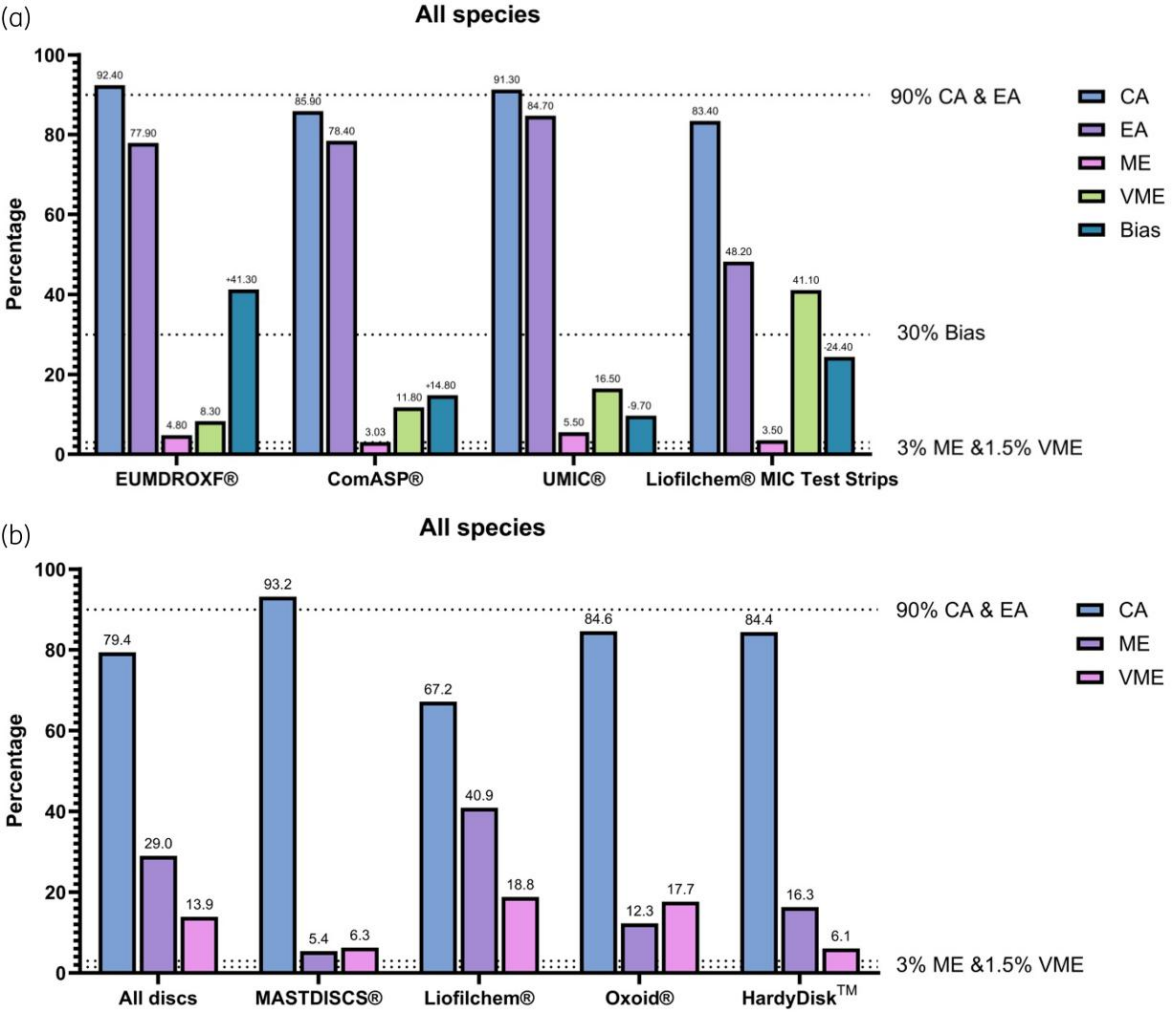
Overall performance of (a) commercially available BMD panels and gradient diffusion test and (b) disc diffusion testing for cefiderocol susceptibility across all species. All biases are presented as positive bias for visualization purposes.

**Figure 2.**
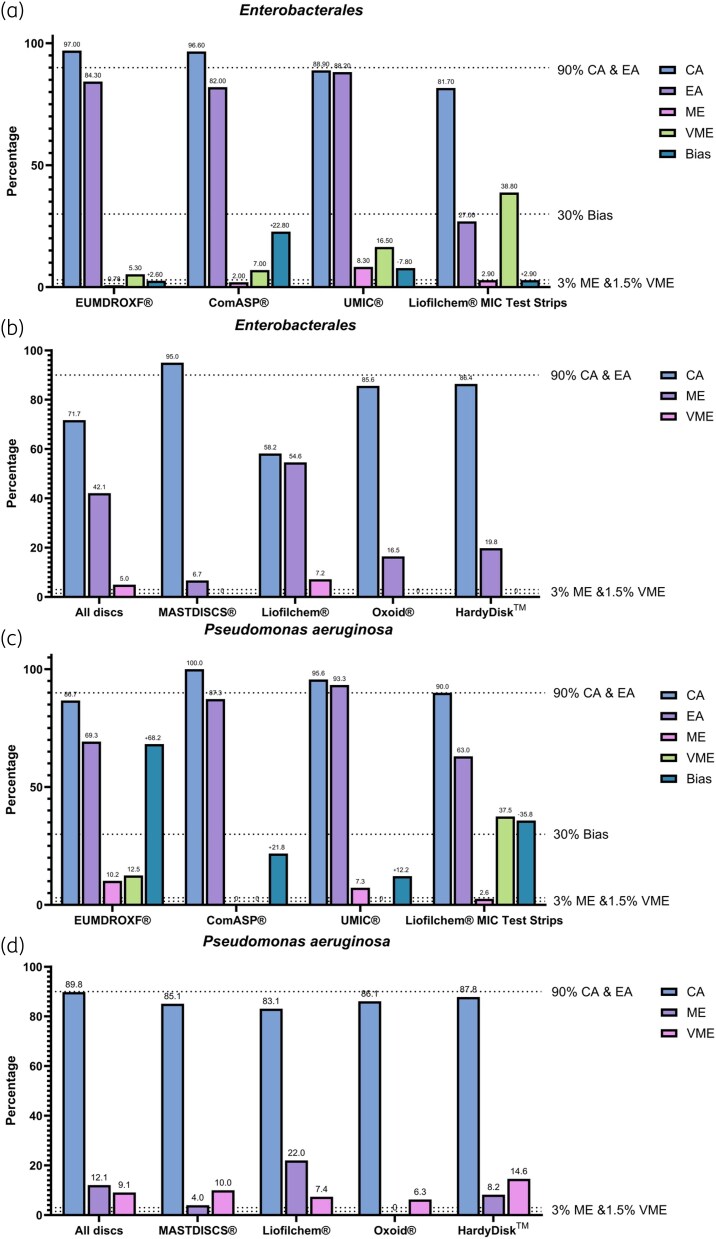

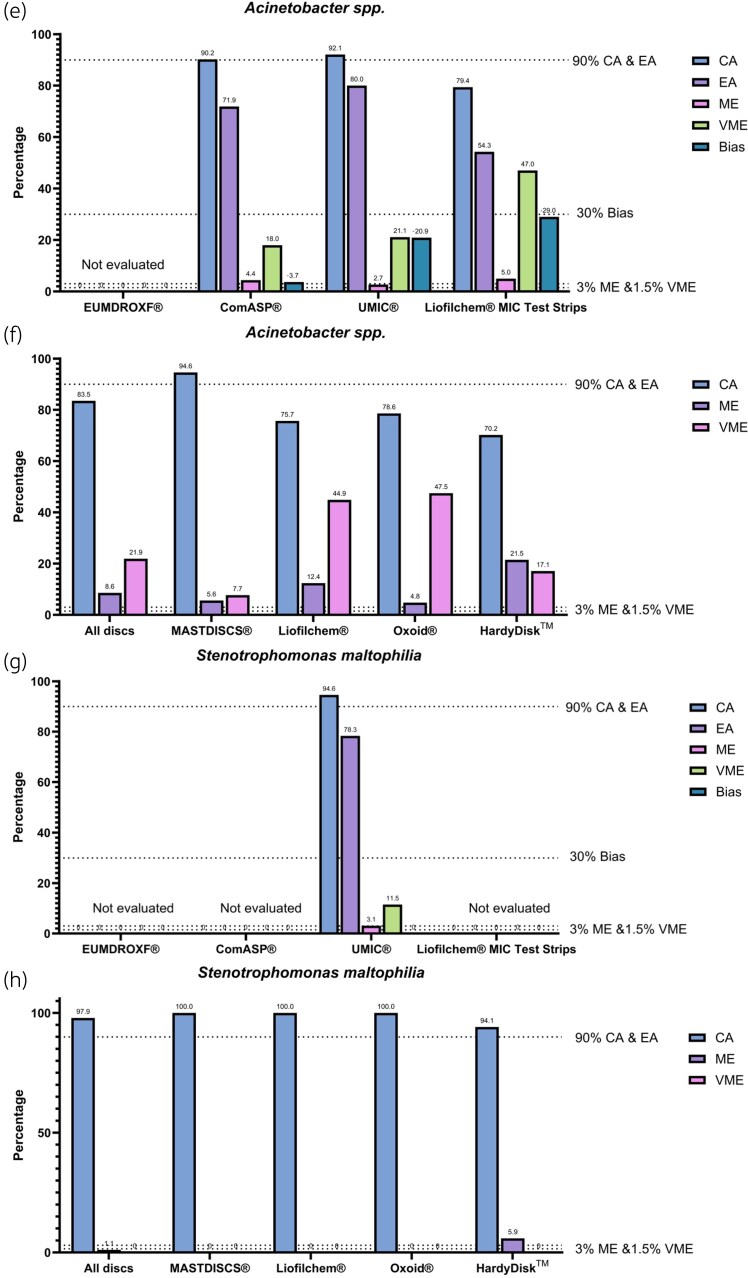
Overall performance of each species for each method: (a) Enterobacterales BMD panels and gradient diffusion test, (b) Enterobacterales disc diffusion testing, (c) *P. aeruginosa* BMD panels and gradient diffusion test, (d) *P. aeruginosa* disc diffusion testing, (e) *Acinetobacter* spp. BMD panels and gradient diffusion test, (f) *Acinetobacter* spp. disc diffusion testing, (g) *S. maltophilia* BMD panels and gradient diffusion test and (h) *S. maltophilia* disc diffusion testing for cefiderocol susceptibility. All biases are presented as positive bias for visualization purposes.

Collectively, the BMD panels represent the most reliable approach, with acceptable CA and low error rates across all tests. The UMIC^®^ panel exhibited a higher CA compared with ComASP^®^, achieving the highest EA rate with minimal bias, establishing it as the most reliable cefiderocol AST among the methods evaluated. However, EUCAST has issued a warning regarding the use of ComASP^®^ and UMIC^®^ BMD panels for cefiderocol susceptibility testing, noting that issues still persist, and further validation is required pending the completion and analysis of an ongoing EUCAST evaluation.^59^ While the EUMDROXF^®^ panel achieved the highest CA, it demonstrated a markedly positive bias and has since been withdrawn from the market. The DD method, led by MASTDISCS^®^, closely mirrors BMD performance, while Liofilchem^®^ discs performed the poorest among DD options, with elevated ME and VME rates. The gradient diffusion method, represented by the Liofilchem^®^ gradient diffusion test, provided moderate CA but was notably hindered by its high VME rate, making it less effective than both BMD and most DD tests. The performance of each method, as derived from all available studies, is presented in Figure 1. The performance of each method for individual microorganisms is illustrated in Figure 2.

## Laboratory challenges in reading and interpreting cefiderocol susceptibility results

The reference method for determining cefiderocol susceptibility is BMD, which requires the use of iron-depleted media and may pose difficulties for diagnostic laboratories.^35^ The requirement for iron depletion significantly affects the reproducibility of the reference method, making it difficult to compare results across different studies. These challenges are further compounded by issues with BMD interpretation, particularly trailing endpoints. Trailing refers to the presence of multiple wells exhibiting minimal growth relative to the positive control.^34^ A study experienced the trailing effect in one-fifth of *A. baumannii* isolates, while another study observed it in the majority of isolates of the same species.^6,34^ In such cases, trailing should be disregarded, and the MIC should be determined at the first well showing substantially decreased growth.^60^

The DD method also introduces additional complexities when interpreting cefiderocol susceptibility. This includes isolates falling within the ATU or the presence of microcolonies within the inhibition zone.^61^ A study proved that when isolates with ZD within the ATU were retested using the reference BMD method, all were found to be susceptible.^6^ This indicates that isolates in the ATU should be further evaluated using an alternative method before being classified as resistant, as recommended by EUCAST.

Additionally, microcolonies, particularly in *Klebsiella pneumoniae*, have been observed, leading to discrepancies in ZD interpretation.^61^ In one study, these microcolonies resulted in 23% more isolates being classified as either within the ATU or as resistant.^61^ Lastly, the lack of consistent cefiderocol breakpoints among EUCAST and CLSI/FDA can result in conflicting outcomes, especially when treating a patient with limited therapeutic choices.^62^

## Limitations of the study

A key limitation of this review is the variability in the types of isolates examined across the included studies, covering Enterobacterales, *P. aeruginosa*, *Acinetobacter* spp. and *S. maltophilia*. This heterogeneity in isolate types may confound direct comparisons between assays, as differences in microbial characteristics could influence test performance outcomes such as CA, EA, ME, VME and bias. Consequently, interpreting and comparing the efficacy of these commercially available tests across diverse isolates should be approached with caution.

## Conclusions

Cefiderocol, a siderophore cephalosporin, employs a novel mechanism by utilizing bacterial iron transport systems to penetrate Gram-negative pathogens, demonstrating potent activity against MDR strains. However, resistance remains a concern, particularly among MBL producers, such as NDM-producing Enterobacterales, where resistance rates can be significant.

Accurate AST for cefiderocol is challenging because it requires ID-CAMHB in BMD methods, which are complex and not routinely feasible in many clinical laboratories. Alternative commercial AST methods, including DD from various manufacturers, have shown variable performance, with discrepancies in susceptibility rates, EA and CA, especially for certain pathogens such as *A. baumannii.* To add to the complexity, EUCAST and CLSI provide differing breakpoints and recommendations for cefiderocol AST, leading to inconsistencies in how susceptibility is classified across laboratories.

While this review focuses on the technical and methodological challenges of AST, it is important to consider the potential clinical implications of AST variability. Although direct outcome data for cefiderocol are limited, the MERINO trial provides a well-documented example.^63^ In this randomized clinical study, patients with ESBL-producing Enterobacterales bloodstream infections treated with piperacillin/tazobactam (deemed susceptible by local laboratories) experienced significantly higher mortality compared with those treated with meropenem.^63^ Discrepancies in AST results and undetected resistance mechanisms contributed to this outcome.^64^ This case highlights how limitations in AST accuracy can lead to suboptimal therapy decisions and adverse patient outcomes, particularly in severe infections.

The standardization and validation of cefiderocol AST methods are critical to ensure reliable susceptibility results, ultimately guiding effective clinical use of this last-resort antibiotic in patients who are most likely to benefit. To achieve this, the highest priority is the standardization of iron depletion conditions and the development of standardized AST panels, which would allow consistent comparisons across methods and facilitate future multicentre studies and initiatives to harmonize breakpoints.

## Transparency declarations

I.B. reports investigator-initiated research funding from Shionogi B.V. for research on Gram-negative infections outside the submitted work; honoraria from Shionogi B.V., Menarini UK and Tillotts Pharma; and participation in advisory boards for GSK and Eumedica. All other authors declare that they have no known competing financial interests or personal relationships that could have appeared to influence the work reported in this paper. L.G. reports investigator-initiated research funding from Shionogi B.V., Pfizer and Advanz Pharma.
